# Advancements and Challenges in the Application of Artificial Intelligence in Surgical Arena: A Literature Review

**DOI:** 10.7759/cureus.47924

**Published:** 2023-10-29

**Authors:** Reda H Mithany, Samana Aslam, Shenouda Abdallah, Mark Abdelmaseeh, Farid Gerges, Mohamed S Mohamed, Mina Manasseh, Andrew Wanees, M Hasaan Shahid, Mahmoud Saied Khalil, Nesma Daniel

**Affiliations:** 1 Laparoscopic Colorectal Surgery, Kingston Hospital National Health Service (NHS) Foundation Trust, Kingston Upon Thames, GBR; 2 General Surgery, Lahore General Hospital, Lahore, PAK; 3 Surgery, Jaber Al-Ahmad Hospital, Kuwait, KWT; 4 General Surgery, Faculty of Medicine, Assuit University, Assuit, EGY; 5 General and Emergency Surgery, Kingston Hospital National Health Service (NHS) Foundation Trust, Kingston Upon Thames, GBR; 6 Orthopaedics, King’s College, London, GBR; 7 General Surgery, Torbay and South Devon National Health Service (NHS) Foundation Trust, Torquay, GBR; 8 General Surgery, Dar El-Salam Hospital, Cairo, EGY; 9 Surgery, Glangwili General Hospital, Carmarthen, GBR; 10 General Practice, Primary Care Polyclinics, Riyadh, SAU; 11 Medical Laboratory Science, Ain Shams Hospital, Cairo, EGY

**Keywords:** ai and machine learning, artificial intelligence education, artificial intelligence and robotics in healthcare, robotic-assisted surgery, artificial intelligence

## Abstract

This literature review delves into the transformative potential of artificial intelligence (AI) in the field of surgery, exploring its evolution, applications, and technological advancements. AI, with its ability to mimic human intelligence, presents a paradigm shift in surgical practices. The review critically analyzes a broad range of research, encompassing machine learning, deep learning, natural language processing, and their diverse applications in preoperative planning, surgical simulation, intraoperative guidance, and postoperative analysis. Ethical, legal, and regulatory considerations, as well as challenges and future directions, are also explored. The study underscores AI's ability to revolutionize surgical visualization and its role in shaping the future of healthcare.

## Introduction and background

As artificial intelligence (AI) continues to evolve, its role in medicine is expected to become increasingly prominent. The ongoing collaboration between clinicians, data scientists, and technologists promises to unlock new frontiers in healthcare. The transformative potential of AI in surgery represents not only a testament to the progress made in the field but also a glimpse into the future of healthcare, where advanced technologies work hand in hand with medical expertise to deliver superior patient care [1].

Artificial intelligence (AI) has a profound history of influencing various medical disciplines. Its origins can be traced back to the mid-20th century when pioneers in computer science conceived the idea of machines capable of emulating human cognitive functions. This audacious vision gave birth to the field of AI, initiating decades of dedicated research and inventive exploration [2].

In the early stages, AI was largely an academic pursuit, with researchers exploring the fundamental concepts of machine learning and pattern recognition. As computing power grew, so did the capabilities of AI systems. In the 1970s and 1980s, AI began to find its place in the medical domain, albeit in more rudimentary forms. Early applications focused on rule-based systems designed to assist with diagnostic decision-making. These systems were limited by the complexity of medical data and the computational resources available at the time [3].

However, as computing technology advanced, so did the potential of AI in medicine. The advent of expert systems in the 1980s marked a significant milestone. These systems, designed to emulate human expertise in specific domains, found applications in fields like radiology and pathology. They provided valuable decision support to healthcare professionals, aiding in the interpretation of medical images and diagnostic data [4,5].

The 1990s witnessed further progress with the emergence of machine learning algorithms that could adapt and improve over time. This was a pivotal moment for AI in medicine, as it enabled systems to learn from large volumes of medical data, uncovering intricate patterns and relationships. These advancements paved the way for more sophisticated applications, including natural language processing for analyzing clinical notes and text-based medical literature [6,7].

In recent years, the convergence of big data, advanced algorithms, and powerful computing resources has propelled AI to the forefront of healthcare innovation. Today, AI is poised to revolutionize surgical practices across various specialties. The integration of AI into surgical workflows holds the promise of significantly enhancing patient care and outcomes. By leveraging AI's capabilities, surgeons can achieve higher levels of diagnostic accuracy, optimize treatment strategies, and execute procedures with unprecedented precision [8].

This article aims to explore the integration of artificial intelligence (AI) in surgery, focusing on its applications, advancements, and potential to enhance surgical procedures and improve patient outcomes. The objective is to provide insights into various AI types, their relevance in surgical practice, and their role in reshaping the future of surgical procedures. Through a synthesis of existing research and scholarly works, the article aims to contribute to a comprehensive understanding of AI's transformative potential in surgery.

## Review

**Figure 1:**
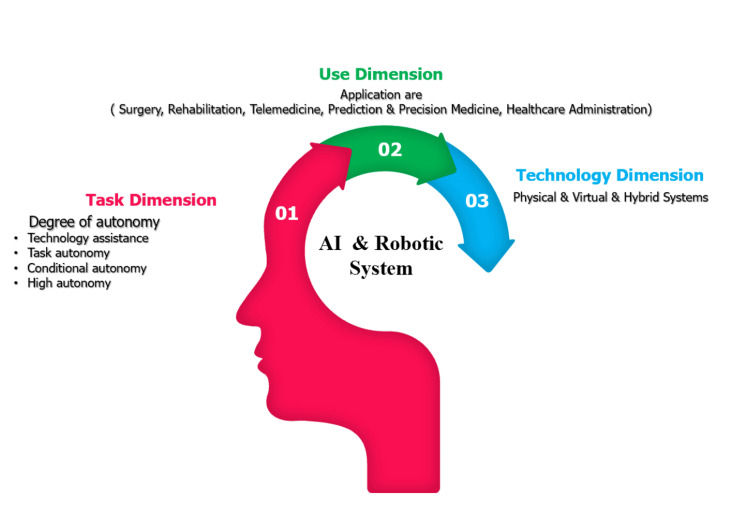
Classification of healthcare systems utilizing AI and robotics AI: Artificial Intelligence This figure was created by one of the authors of this article.

The integration of artificial intelligence (AI) into the field of surgery holds substantial significance (Figure 1). Surgeons, leveraging their unique expertise, are poised to play a pivotal role in this transformative process. Collaboration with data scientists is essential, as it enables comprehensive data capture throughout the care process, offering vital clinical context. AI exhibits the potential to revolutionize surgical education and practice, promising a future where procedures are optimized for delivering the highest quality patient care. This synergy between surgeons and AI technology marks a paradigm shift in surgical techniques, promising more efficient and effective procedures [7,9].

AI has become increasingly integral in various surgical specialties, revolutionizing approaches to patient care and outcomes. This article delves into the current applications of AI across different surgical disciplines, showcasing its potential to refine diagnostic accuracy, treatment strategies, and overall surgical precision [10].

**Figure 2:**
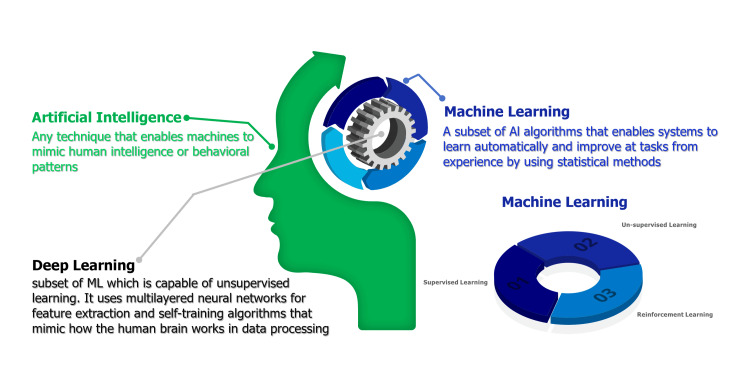
Differences between AI, ML, and DL AI: Artificial Intelligence; ML: Machine Learning; DL: Deep Learning This figure was created by one of the authors of this article.

The field of surgical technology and AI is divided into three key areas: preoperative planning, intraoperative guidance, and surgical robotics. Preoperative planning focuses on the requirements of accuracy, automation, privacy, and speed in preparing for surgery. However, it faces challenges related to multi-modal input, patient diversity, complicated symptoms, and less informative diagnostic data. Sub-areas within preoperative planning include classification, detection, segmentation, and registration. Intraoperative guidance demands real-time precision and high-resolution data but encounters difficulties in limited and low-resolution input, tissue deformation, and environmental variance. Sub-areas of intraoperative guidance encompass 3D shape instantiation, endoscopic navigation, tissue feature tracking, and augmented reality. Surgical robotics, with requirements for precision, robustness, real-time performance, and safety, deals with challenges related to accurate kinematics and dynamics modeling, perception of the dynamic environment, and high autonomy. Sub-areas in surgical robotics include robotic perception, localisation and mapping, system modelling and control, and human-robot interaction [11,12] (Figure 2).

AI plays a significant role in these areas, employing various learning methods such as supervised learning, unsupervised learning, semi-supervised learning, learning from demonstration, reinforcement learning, federated learning, and meta-learning. AI can enhance preoperative planning by improving diagnosis, optimizing surgical plans, and ensuring patient privacy. In intraoperative guidance, AI aids surgeons with real-time information, precise navigation, and augmented reality overlays to enhance surgical outcomes. In surgical robotics, AI-driven systems offer more accurate and safer procedures, advanced control and manipulation, and improved human-robot collaboration [13].

Application of AI in different surgical specialties

**Figure 3:**
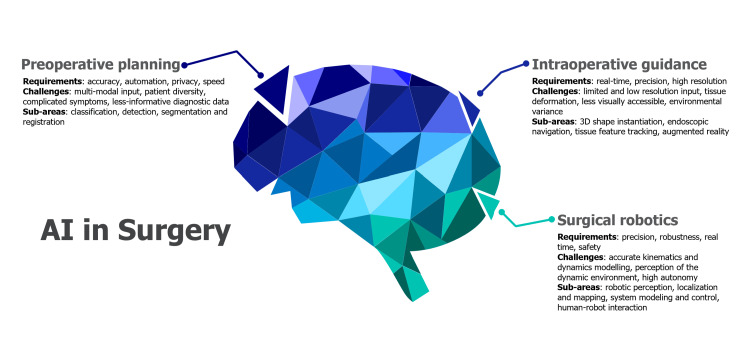
AI in Surgery AI: Artificial Intelligence This figure was created by one of the authors of this article.

The application of AI in various surgical specialties has been a groundbreaking development in the field of healthcare. AI technologies are increasingly being integrated into surgical practices across different specialties, offering improved precision, efficiency, and patient outcomes (Figure 3).

In colorectal surgery, AI offers several advantages including improved diagnostic accuracy through the analysis of genetic data, enabling early detection and personalized treatment strategies. It facilitates non-invasive screening by assessing individual risk profiles, reducing the need for invasive procedures such as colonoscopy. AI aids in real-time polyp detection during colonoscopy, enhancing the effectiveness of this screening method and also optimizes virtual colonoscopy by improving the detection of colorectal polyps. Furthermore, AI automates the analysis of capsule endoscopy images, reducing interpretation errors and expediting lesion detection. It can also enhance blood tests by identifying biomarkers associated with colorectal cancer (CRC) for early detection and risk assessment. AI-based algorithms assist in predicting patient prognosis, guiding treatment decisions, and identifying potential drug targets. Additionally, AI contributes to the reduction of interval cancers by improving the detection of flat or small polyps. At a population level, AI helps assess the risk of colorectal cancer, enabling more efficient screening strategies and resource allocation [14].

AI-driven systems offer significant benefits in polyp detection, enhancing sensitivity in CRC screening and reducing the rate of missed post-colonoscopy CRC cases, especially for right-sided, flat, or small polyps. Furthermore, AI models contribute to polyp characterization, aiding in distinguishing neoplastic lesions from non-neoplastic mimickers and providing insights into submucosal invasion depth. Various imaging techniques, including magnification endoscopy, endocytoscopy, confocal laser endomicroscopy, magnifying chromoendoscopy, autofluorescence endoscopy, and white light endoscopy, can be combined with AI to improve the precision of polyp characterization. Additionally, AI plays a role in treatment strategies by assisting in robotic-assisted surgery and chemotherapy, offering more efficient and minimally invasive surgical options, as well as personalized drug treatments for CRC patients. Lastly, AI contributes to the field of precision oncology, helping identify genetic biomarkers, drug interactions, and potential therapeutic targets, thus tailoring treatment approaches to individual CRC patients more effectively [14,15].

Similar to colorectal surgeries, upper gastrointestinal surgeries also benefit from AI technologies, particularly in the diagnosis and treatment of conditions such as esophageal cancer. AI algorithms can analyze endoscopic images and help detect abnormalities or early-stage malignancies, enabling timely interventions. Additionally, AI systems play a role in surgical planning, ensuring optimal approaches to resection and minimizing complications. In hepatobiliary surgery, AI aids in liver disease diagnosis, predicting patient outcomes, and optimizing liver resections. Machine learning models analyze liver imaging data to determine tumor margins and guide surgeons during the resection process. These AI-driven insights lead to better surgical outcomes and postoperative patient care [16-18].

In the field of neurosurgery, AI is employed in image-guided surgery, assisting surgeons in navigating through intricate brain structures with higher precision. Machine learning algorithms help in real-time tumor segmentation, aiding surgeons in the removal of brain tumors while minimizing damage to healthy tissues. Additionally, AI-powered diagnostic tools can enhance the detection of neurological disorders, such as strokes, by rapidly analyzing medical imaging data [19,20].

In the realm of orthopedic surgery, AI plays a pivotal role in customizing treatment plans. AI algorithms assist in predicting patient outcomes after joint replacements, allowing for personalized care. Furthermore, robotic surgical systems are increasingly utilized in orthopedics, enhancing the precision of procedures like joint arthroplasty. These systems offer real-time feedback to surgeons, helping them make more informed decisions before and during surgery [21].

In the field of cardiology, AI applications have brought about significant advancements in the diagnosis and treatment of heart-related conditions. Machine learning models can analyze cardiac images, such as echocardiograms and angiograms, to detect anomalies and assess heart function. AI-powered monitoring devices can continuously track patients' cardiac health and provide early warning signs, leading to improved management of heart conditions. Moreover, during cardiac surgeries, AI-driven robotic systems aid surgeons in performing complex procedures with a high degree of accuracy, reducing the risk of complications [22,23].

The use of robotic-assisted keyhole surgery in gynaecology has expanded due to technical advancements, offering benefits like improved depth perception, reduced tremor, increased precision, faster learning curve, and enhanced surgeon comfort compared to traditional keyhole and open surgeries. Robotic-assisted surgery improves performance without increasing operating time, minimizes blood loss and complications, and reduces the need for open surgery. Surgeons using robots experience fewer musculoskeletal issues, making it a safer and more effective option for complex gynaecological procedures, especially for patients with high BMI or respiratory problems. This innovation has decreased the need for open surgeries, lowering the risk of conversions, impacting cost-effectiveness considerations [24].

**Figure 4:**
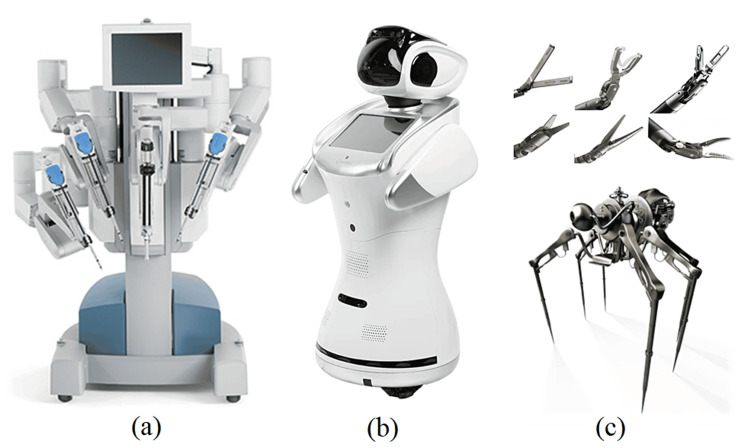
Robotics in surgery (a) Davinci; (b) AI Robot; (c) Nanorobots This figure was created by one of the authors of this article.

The rapid strides in robotics including Davinci, AI robots and nanorobotics (Figure 4) and their wide-ranging applications, from enhancing materials like paint coatings to revolutionizing medical treatments, underscore the intersection of electronic fabrication and particle systems. This convergence enables the seamless integration of nanocomputational systems into a variety of materials. Moreover, recent advancements in controlling mechanisms within biological systems highlight the imperative for further exploration in areas such as cell signaling and precise treatments. Within the interdisciplinary realm of nanorobotics, particular attention is given to its medical applications, encompassing target identification, drug delivery, and minimally invasive surgical procedures. These innovations have given rise to autonomous nanorobots capable of harnessing blood glucose for energy, detecting cancer cells, and effectively treating diseases [25].

Alongside all the benefits of AI, the adoption of artificial intelligence (AI) in healthcare brings forth several concerns and negative impacts. These include challenges related to data collection, as healthcare data is often siloed and access is limited, posing obstacles to effective AI development. Data privacy and security are significant concerns, with the potential for breaches of sensitive patient information. Bias in AI algorithms, overfitting, and data quality issues can lead to distorted or unfair outcomes, exacerbating healthcare disparities. The "black-box" nature of many AI systems presents transparency and accountability challenges. Ethical concerns revolve around responsibility and standards for AI decision-making, while job displacement fears among healthcare professionals persist. Addressing these concerns requires improved data sharing, security measures, bias mitigation strategies, transparency, ethical frameworks, and empirical validation of AI applications [26].

This overview underscores the promising applications of AI across various surgical specialties. It is important to acknowledge that the field is continuously evolving, and further research and technological advances will expand the scope and impact of AI in surgery.

## Conclusions

Artificial intelligence has emerged as a pivotal force in surgery, promising to enhance patient outcomes and transform traditional surgical approaches. The review has highlighted the significant impact of AI in various surgical domains, spanning from preoperative planning to postoperative analysis. Machine learning and deep learning algorithms, coupled with natural language processing, showcase immense potential in predicting surgical outcomes, aiding decision-making, and improving surgical education. Additionally, AI's integration into surgical visualization through advanced technologies like virtual reality and three-dimensional (3D) printing offers unparalleled opportunities for surgical training and planning. However, challenges such as generalizability, cost-effectiveness, and ethical considerations remain, necessitating continued research and collaboration to maximize the benefits of AI in surgical practice. Overall, the evolution and application of AI in surgery hold the promise of reshaping the landscape of healthcare, ushering in a new era of precision, efficiency, and superior patient care.
